# The Process of Integrating Family Planning Services with Other Reproductive Health Services in Low and Middle-Income Countries: A Scoping Review

**DOI:** 10.5334/ijic.8912

**Published:** 2025-07-01

**Authors:** Farina Gul, Zohra S. Lassi, Gizachew A. Tessema, Jawaria Mukhtar Ahmed, Mohammad Afzal Mahmood

**Affiliations:** 1 School of Public Health, University of Adelaide, Adelaide, Australia; 2 Robinson Research Institute, University of Adelaide, South Australia, Australia; 3 Curtin School of Population Health, Curtin University, Australia; 4 Department of Community Health Sciences, Aga Khan University, Karachi, Pakistan; 5 Faculty of Medicine, Universitas Indonesia, Surabaya, East Java, Indonesia

**Keywords:** family planning, reproductive health, integration, low and middle-income countries

## Abstract

**Introduction:**

In low-resource settings, fragmented family planning (FP) services limit contraceptive access, contributing to high unmet needs and unintended pregnancies. Integrating FP with existing reproductive health services can improve access and continuity of care. This review examines the integration strategies in low and middle-income countries (LMICs).

**Theory and Methods:**

We conduct a scoping review across five databases for peer-reviewed literature and Google Scholar for grey literature, guided by Joanna Briggs Institute (JBI) and Arksey & O’Malley’s frameworks. Data were charted study characteristics and details of integration process. Results were reported following PRISMA-ScR guidelines.

**Results:**

The total of 37 studies from LMICs were included. Only five studies provided explicit definitions of integration. Key strategies involved aligning FP with other services, promoting dual-method use in HIV care, and incorporating long-acting reversible contraception with post-abortion and postpartum care. Training was provided to various health workers to support services integration. Most Models used co-location of services within the same facility. Innovative approaches, such as; the Happy Client Model and private counselling spaces. Integration was influenced by provider skills, workload, communication dynamics, training gaps, and supply constraints.

**Conclusion:**

A review identified diverse methods and factors for integrating family planning services. Clear operational definitions and innovative services delivery models are critical for effective integration. Further research should inform context-adaptable frameworks for implementation in resource-constrained settings. These findings can inform integrated care policy by highlighting the need for coordinated service models, provider training, and context-sensitive strategies to optimise FP access in LMICs.

## Introduction

In low and middle-income countries (LMICs), over 200 million women lack access to modern contraception, contributing to high level of unintended pregnancies [1]. The health system in these countries faces numerous challenges in providing family planning (FP) services, including limited contraceptive options, inadequate infrastructure, stock-outs, and workforce shortage [2,3]. Consequently, women often miss opportunities for FP counselling even when accessing other reproductive health services, for example, studies from Ethiopia and Kenya have shown that postpartum women were discharged without receiving any information or counselling about contraceptive options during antenatal or postnatal care visits [4,5,6].

Integrating FP services with broader reproductive health services can improve access and continuity of care, especially in low-resource settings [7]. Definitions of integration vary: in some contexts, it refer to linking care and treatment sectors, while in health systems, it entails coordinated management of care to ensure seamless service delivery across time an disciplines [8,9]. Integration levels range from minimal collaboration, to full co-location of services provided by a single provider or within the same facility [10]. At its core, integration fosters collaborative care through clinical and professional coordination, underpinned by shared roles, accountability and person-focused care pathways [11], while professional integration relies on shared competencies, roles, responsibilities and accountability to deliver a comprehensive continuum of care [12].

Effective integration relies on referral system and task shifting, enabling trained non-specialists to deliver FP services. Approaches vary by services type and complexity [13,14,15]. Integration strategies differ by service context; for instance, FP with HIV care emphasizes infection prevention, while with abortion care, it focuses on preventing repeat unintended pregnancies [16]. Administrative process, such as documentation and record-keeping, are also integral to services alignment [17]. The integration can occur at multiple level: micro (clinical), meso (organisational), and macro (policy/governance), each requiring different mechanisms of coordination and support [11].

Previous reviews [18,19,20] have focused on outcomes of FP integration, such as increased contraceptive use or reduce unmet needs (defines as the proportion of women who want to avoid or delay pregnancy but are not using any method of contraception), but have not explored the integration process itself.. This scoping review addresses the gap by examining how FP services are operationally integrated with reproductive health services, including antenatal care (ANC), postnatal care (PNC), post-abortion care (PAC), HIV/AIDS, and immunisation services. these platforms represent critical touchpoints for expanding FP access during routine healthcare encounters [21].

By mapping strategies, processes, and enabling factors for integration, this review provides insights into how FP services can be more effectively embedded within reproductive health system in LMICs. It aims to inform policies and programs seeking to optimise service delivery and reduce missed opportunities for FP counselling and provision.

## Methods

This study adopts the framework proposed by Arksey & O’Malley [22]. The review protocol has been registered with the Open Science Framework database (registration DOI: https://doi.org/10.17605/OSF.IO/Z73QP) [23], and followed five steps summarised below.

### Step 1: Identifying the review question

This study aims to understand the integration process for FP services with other reproductive health services. For this review, “integration” is defined as providing a continuum of care through coordinated services across different levels and sites of care tailored to individuals’ needs through their life course [24]. A “process” of integration refers to a series of organisational actions or activities intended to integrate patient care services into a single process across people, functions, activities, and operating units over time, ensuring that care is delivered in a cohesive and coordinated manner [25,26].

The primary question for this review is:

What are the integration processes among the initiatives that integrate FP services with other reproductive health services in LMICs?

The review attempts to answer four questions:

1. How did selected studies define integration when combining FP services with other reproductive health services?
2. What strategies have been adopted to integrate FP services with other reproductive health services?
3. What were the factors that impacted the integration process?
4. How did the studies link the integration process with FP outcomes?

Detailed inclusion criteria using the PICCOS framework are provided in Table 1 of Supplementary File.

### Step 2: Identification of literature

The literature search for this review was conducted using both published and ‘grey’ literature. Following the Joanna Briggs Institute (JBI) [27] guidance for a scoping review. The search process involved three steps. First, an initial search was performed using the online database of the University of Adelaide (UoA). Second, a comprehensive search was conducted across five databases- PubMed, Cochrane, CINAHL, Embase, and Web of Science- using identified keyword and index terms. Finally, the grey literature was searched using Google Scholar and key organisational websites, including WHO, FP2030, Guttmacher Institute, Gates Foundation, UNFPA, and USAID. The lead investigator, with support from the research team and a librarian, developed a comprehensive search strategy. The search covered January 2010 to July 2023 and was limited to English language materials.

### Step 3: Study selection

**Figure 1:**
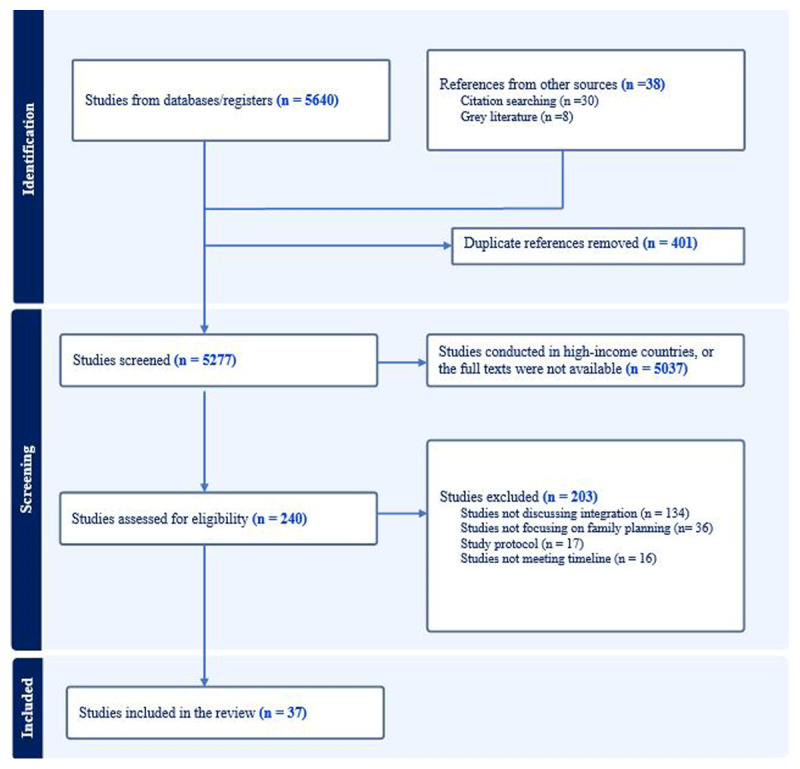
PRISMA-ScR flow diagram.

Two reviewers independently screened titles, abstracts, and full texts, resolving disagreements through discussion or consultation with third reviewer. The selection process adheres to the Preferred Reporting of Items for Systematic Reviews and Meta-Analyses (PRISMA) statement [28]. Figure 1 below illustrates the article selection process. The selection process adheres to the Preferred Reporting of Items for Systematic Reviews and Meta-Analyses (PRISMA) statement [28].

The inclusion criteria were as follows:

1. Intervention studies using any quantitative and qualitative methodologies that address integration.
2. Studies that integrate FP services with existing reproductive health services.
3. Reproductive health services identified in the studies must include HIV/AIDS care, antenatal and postnatal care, postpartum care, post-abortion care, immunisation care, and delivery care.
4. The studies conducted at the facility level only.

The exclusion criteria were:

1. Studies such as commentaries, editorials, and study protocols.
2. Systematic reviews or literature reviews that discuss the uptake of FP without addressing the integration process.
3. Studies discussing only training of human resources without mentioning the integration process.
4. Studies where integration occurs only at the community level or considers integration merely as a referral.

### Step 4: Data charting

The data was extracted using Microsoft Excel software. Lead author developed the data extraction checklist with input from other authors. The checklist included categories such as author, year of publication, study country, methodology used, and study outcomes.

Data were analysed using inductive and deductive thematic analysis. Codes were grouped into themes such as capacity building, referral mechanisms, and client contact approaches. Quotations were used to illustrate facilitators and barriers to integration.

Charting process also identified how selected studies defined integration. Further information on integration processes was charted, including human resource capacity building, referral mechanisms, and approaches used to contact clients and introduce FP services in combination with other services.

Themes were refined collaboratively by the lead authors and co-authors through iterative discussions, reviewing coded data against the research questions and integration frameworks to ensure consistency, accuracy, and relevance to review objectives.

### Step 5: Collating, summarising, and reporting the results

Authors reviewed the extracted evidence individually and cross-verified the results for accuracy. This triangulation process enhanced the validity of the research. The results were summarized into three broader predefined themes: how integration was defined in the studies, the integration processes used, and how integration was linked to FP outcomes.

The review analysed both qualitative and quantitative information simultaneously. The qualitative information, based on the perceptions and experiences of providers and clients, helped identify factors impacting the integration process. Therefore, the sub-category of factors impacting the integration process included qualitative data, incorporating direct quotes from the studies to highlight facilitating and hindering factors.

## Results

The search identified 5,640 records; after screening, 37 [29,30,31,32,33,34,35,36,37,38,39,40,41,42,43,44,45,46,47,48,49,50,51,52,53,54,55,56,57,58,59,60,61,62,63,64,65]. A detailed overview of the characteristics of studies is provided in Supplementary File Table 2.

Studies integrating FP with HIV/AIDS care promoted ‘dual method use’ [29,33,34,37,39,41,44,50,57,62], defined as combining the use of modern contraception methods with a condom to protect against HIV transmission [62]. This was described in several ways: “modern contraception method along with condom” [50], and “using condom plus another contraceptive method” [30,33,34,41,44].

Studies combining FP with immunisation services mainly aimed to increase knowledge or record the intention to use contraceptive methods [40,43,66]. Two studies [35,63] also discussed offering FP services alongside increasing knowledge. Studies integrating FP with post-abortion or postpartum care [31,32,58,64] provided both short-acting and long-acting reversible contraception within the facility.

### How integration was defined in selected studies

Only five [33,40,61,65,66] explicitly defined integration, primarily describing in terms of co-location of services within the same facility, available on the same day or through flexible referral arrangements. These definitions are summarised in Supplementary File Table 3.

### Integration process

The integration process involves clinical and professional activities such as staff training, referral systems, client engagement strategies, and service delivery innovations.

#### Human resource capacity building for integration

Four of the studies [29,39,63,66] focused on strengthening the capacity of existing FP providers by training them to conduct counselling, assess client’s pregnancy risk, and make appropriate referrals for FP services with health facilities. In studies where FP was integrated with HIV services, counsellors and peer educators were equipped [34,41,42,48,50,53] to conduct group counselling, promote dual method usage, and refer clients for contraceptive methods. Three of these studies [41,48,62] trained nurses and other staff to provide FP and contraceptive services. Facilities integrating FP with immunisation services trained vaccinators to educate, counsel, and refer women for FP services. Two of these studies [39,63] provided additional FP screening, pregnancy risk assessment, and training. Studies reviewing post-abortion and postpartum FP integration included training for healthcare providers such as nurses, doctors and midwives to provide long-acting FP services [31,59,64]. Several studies mentioned the type of training material used [29,31,32,39,44,45,47,52,59]. The average duration of training was reported in six of these studies [34,39,44,48,55,59] and typically consisted of three days of training for providers on FP services. Detailed information on provider types, training content, training duration, and materials used across different service contexts is presented in Supplementary Table 4.

#### Referral mechanism and service provision

The studies described two main referral arrangements for integrated FP services: (1) Co-location, where services are offered in different departments or units within the same health facility, requiring internal referral; and (2) same-room provision, where services are delivered within the same physical space, often by the same or different provider, without requiring formal referral. Integration with HIV/AIDS services often involves providing FP methods within the same unit and mostly by the same provider [34,38,41,42,46,50,53]. However, three studies [42,45,46] referred clients outside the facilities for permanent contraceptive methods (sterilisation or vasectomy). Studies integrating FP with immunisation services referred mothers to FP units within the facilities. Additionally, two studies [47,54] discussed FP integration with multiple maternal health services, referring antenatal clients for contraceptive methods within the co-located facility, and providing FP services at the same unit to the postnatal clients.

#### Approaches to contact client for initiating FP service

Studies employed varied strategies to introduce FP services, such as proactive client engagement, provider-initiated counselling, and group education, tailored to service platforms and client contexts. The studies on HIV/AIDS services discussed two approaches: offering FP services in the waiting rooms [37,41,42], and providing group education sessions for women with HIV conducted by peer educators [38,41,50,53]. Similarly, immunisation clinics also utilised education sessions to promote FP services [39,42,43,55]. In contrast, women were approached for FP at various postpartum periods (6–10 days, 6–8 weeks, and 9 months) in the clinics where postpartum services were provided. A study integrating FP with multiple maternal health services [54] employed the Provider-Initiated FP (PIFP) model, wherein service providers initiated discussions about FP methods during the service provision (refer to Table 1).

**Table 1:** Integration Process: Approaches Used to Contact Clients to Initiate FP Services.


AUTHORS (REF)	CLIENT ENGAGEMENT APPROACH	SERVICE PROVISION APPROACH

**Integration with HIV**		

Baumgartner et al., 2014 [29]	Clients screened for unintended pregnancy and counselled during HIV visits.	FP offered via co-located HIV care

Demissie and Mmusi-Phetoe, 2021 [37]	Health workers counselled women in HIV clinic waiting areas.	Referral to FP unit within same facility.

Dulli et al., 2019 [38]	Peer educators informed clients about dual methods and FP availability.	Clients received same-day FP services.

Grossman et al., 2013 [41]	Group talks conducted in waiting areas by peer educators.	Clients accessed FP services within clinic.

Hawkins et al., 2021 [42]	Announcements made during HIV care visits for FP availability.	Referral to FP provider within clinic for unavailable methods.

Joshi et al., 2016 [44]	Staff assessed fertility desire and referred eligible women.	FP services provided by trained providers.

Malama et al., 2020 [48]	Staff from another department (trained to promote services) generate demand for FP	The client, with the invitation, visited the clinic and availed of services.

Medley et al., 2022 [50]	Counsellors initiated fertility discussions during health talks.	Nurses provided FP in private HIV clinic spaces.

Mudiope et al., 2017 [53]	FP champions provided group and individual education.	Referrals made to co-located FP services.

Thyda et al., 2015 [62]	Screening for FP needs in waiting rooms.	Referral to FP counselling rooms.

**Integration with Postabortion and postpartum care**		

Belemsaga et al., 2018 [64]	Integrated postpartum care scheduled with immunisation visits (days 6–10, 6–8 weeks, 9 months).	—

Pradhan et al., 2019 [58]	Counselling for PPIUD offered during ANC/PNC and post-delivery.	PPIUD provided post-delivery upon consent.

Tawfik et al., 2014 [60]	Counselling in separate room for woman and family.	Referral to FP service near postpartum ward.

**Integrating FP with Immunisation services**		

Cooper et al., 2020 [35]	Same-day education provided by community-level assistants.	FP services/referrals offered by nurses.

Cooper et al., 2015- [66]	Vaccinators used job aids for one-on-one messaging.	Referral to co-located FP room; same-day services provided.

Dulli et al., 2016 [39]	FP education via pre-immunisation talks.	Referrals to FP provider for counselling and method choice.

Erhardt-Ohren et al., 2020 [40]	Referral cards distributed during immunisation wait time.	Same-day FP services at same facility.

Ijarotimi et al., 2023 [43]	FP education at each infant vaccination visit (group/individual).	Referral to FP clinic same day or later.

Nelson et al., 2019 [55]	Brief FP discussion and referral during immunisation.	Tracking referrals and leaflet distribution.

Vance et al., 2014 [63]	Vaccinators screened for pregnancy risk with job aids.	Referral to co-located FP services.

**Integrating FP with Maternal Health services**		

Mutisya et al., 2019 [54]	PIFP model: providers initiated FP discussions during HIV care.	FP method offered by same provider.

Mackenzie et al., 2018 [47]	Multiple provider interactions in ANC/PNC settings.	FP provided in ANC or PNC by designated staff.

Memon et al., [52]	Establishment of private FP counselling corners.	—


### Novel Approaches used in the integration

One study in Zimbabwe [48] utilised the Happy Client model to generate demand for integrated FP services by identifying satisfied users of long-acting reversible contraception (LARC) and training the to share their positive experiences with other clinic visitors in waiting areas during community outreach activities, thereby encouraging uptake of FP methods. Similarly, a study from Uganda [53] employed FP Champions, HIV-positive mothers who had disclosed their status to at least one confident, had prior experience working in HIV clinics, and held a positive attitude towards FP. These clients advocate LARC among HIV-positive women by sharing their personal experiences. Additionally, a study from Afghanistan [60] established a private counselling space to address cultural barriers, while another study in Pakistan [52] renovated an existing room and repurposed it as a private “counselling corner”.

### Factors impacting the integration process

The factors impacting the integration process were discussed in studies using mixed methods or qualitative approaches [43,54,56,61,66]. Some factors enabled the integration process. For instance, training provider increased their confidence in offering FP services. A provider in one study [43] noted, “*About 50–60 mothers bring their babies daily for vaccination. Integrating FP education into the vaccination visits will…. make more women decide to do FP”*. Additionally, studies identified the need to train more staff for FP services to accommodate the larger number of clinical visitors, as a lack of trained staff leads to delays in integrating services and loss of potential clients. A provider in one of the studies [61] commented, “*I think…. you need more staff; I alone cannot do all this work… the same days, I have to do postnatal consultations, weigh the children, perform the BCG, etc. If we have to integrate nutrition, it becomes too much*.” The lack of re-training was also highlighted in a study [43],” *Yes, I have some experience in FP, but my last training in FP was in 1995”*.

Providing services on the same day and at the same location was a facilitating factor, although no study recorded health system perspectives by including providers’ views on this approach. One provider in a study [61] discussed the potential advantage of providing same-day services *“…women who disappear after delivery….they don’t come back anymore. So, as soon as they come to deliver, if we take advantage of offering them all these services*”.

Lack of privacy while informing about FP services at the clinic was identified as a barrier. FP counselling and services offered in open areas were discouraged by providers. One provider [56] mentioned, “*[At] times the clinicians are two in a room and … maybe some clients feel that FP is something private and confidential … so if there is a man and there is a woman … the other person ends up not talking about it [and] then goes home not satisfied”*.

Key enabling and constraining factors influencing the integration process across studies are summarised in Supplementary Table 5.

### Linking FP outcomes with the integration process

The primary objective of six studies was to increase the use of dual contraceptive methods through integration [33,37,38,44,50,62]. These studies employed training, counselling, and referral mechanisms to promote dual methods, with four of them [37,38,44,50] achieving this outcome. Studies aimed to increase the uptake of contraception [31,53,58,63] focused on counselling and promoting FP. Three studies [29,32,48] aimed to reduce unmet needs and utilised training, referrals, counselling, and co-location.

Three studies targeted the reduction of unintended pregnancies; two [34,41] used counselling and group-based information to achieve this goal. However, one study [46] did not discuss the integration process and observed no impact on unintended pregnancies. The ways in which integration strategies were linked to FP outcomes—such as increased contraceptive uptake, dual method use, and reduced unmet need—are summarised in Supplementary Table 6. Additionally, three studies [30,39,43] aimed to measure the effectiveness of integration, with each defining ‘effectiveness’ differently. For example, the study from Botswana [42] measured effectiveness by women’s intentions to use various contraception methods, such as copper plus hormonal IUD as “very effective” and three-month injectable and oral pills as “effective” with no method deemed “ineffective”. The study from Tanzania [29] considered the effectiveness of integrating HIV and FP through the use of dual methods, following the referral process. A study from Kenya [38] measured effectiveness by the number of clients who received FP counselling, attended health sessions and, used FP, and it discussed the integration process in detail.

## Discussion

1. **Defining and operationalising Integration:** The review explored FP integration from a health systems perspective, emphasizing clinical and professional activities [67]. Only five studies defined integration, focusing on operational elements, timing, location, and delivery mechanisms—shaped by setting, cost, and provider type, indicating that integration largely occurs at the clinical level.
2. **Workforce Preparedness and Training Challenges:** Training providers is central to the integration process, with studies highlighting capacity-building across HIV, immunisation, and postnatal care settings to support FP delivery. However, increased provider workload emerged as a recurring challenge [68,69]. Training additional staff and offering regular refresher courses were suggested strategies to sustain integration [70]. These findings underscore the importance of ongoing professional development to ensure effective and efficient delivery of integrated FP services.
3. **Referral Mechanisms and Service Coordination:** Referral mechanisms are an essential component of the integration process. A common approach among the studies reviewed is to provide FP services within the same facility but in a different unit, known as ‘co-location’, allowing clients to access services on the same day. This approach promotes acceptability and increases the uptake of FP services [71]. However, mechanisms for following up with clients for FP uptake were not discussed in the selected studies, except one [29]. Incorporating follow-up mechanisms is crucial for making the integration process smooth and sustainable, as follow-up helps achieve desired outcomes, such as reducing unmet need [72].
4. **Practical Implication for Program Desing:** This review highlights the operational realities of integrating FP services with other RH services in LMICs, offering practical insights into how integration is enacted at the clinical level. It underscores that while training is foundational, systematic factors such as staffing capacity, referral coordination, and privacy in service delivery significantly shape outcomes. These findings hold value for program implementers and policymakers aiming to strengthen reproductive health systems. By synthesising integration strategies across diverse platforms, including HIV care, immunisation, and postpartum services, this review contributes actionable knowledge to the field of health systems integration in low-resource settings.
5. **Contributions to the Literature and Policy Discourse:** The integration of FP services into other reproductive health services offers an important pathway to address structural barriers and reduce missed opportunities for contraceptive provision. Our findings underscore the integration, when operationalised through strategies such as co-location, provider-initiated counselling, and coordinated referrals, can directly improve service uptake and continuity of care. These insights build on previous research highlighting the importance of integrated service delivery models in improving access and client satisfaction in LMICs [71]. The emphasis on co-location aligns with WHO guidance on strengthening primary healthcare through service integration [73], while our findings on workforce burden and the need for refresher training reinforce recommendations from studies in integrated HIV-SRH settings [68,70]. This review contributes a process-oriented perspective that complements outcome-focused literature, helping to bridge the gap between integration policy and operational practice.
6. **Research and Policy Direction:** Based on this review, several next steps are necessary to advance the integration agenda in LMICs. Future implementation research should focus on evaluating how integration strategies can be scaled sustainably within different health system contexts, especially in settings with workforce shortages or weak infrastructure. Cost-effectiveness studies and long-term evaluations are also needed to understand the downstream impacts on contraceptive use, maternal health outcomes, and health system efficiency. Importantly, participatory approaches involving service users, particularly women, adolescents, and marginalised groups, should be prioritised to ensure that integrated services are culturally responsive and client centred. Lastly, policy frameworks must support adaptive service models that allow flexibility in implementation while maintaining fidelity to rights-based person-centred care.
7. **Limitations:** This scoping review has certain limitations. Firstly, defining the integration process is challenging because it involves integrating patient care across various functions and activities [74]. Without a clear checklist of activities, it is difficult to determine if this review covered all aspects of process integration. Additionally, the review did not address the level of integration in terms of partial (services provided at different facilities) or complete (where all aspects of services are integrated) integration [75]. The selected studies focused on service delivery integration without explaining professional and administrative integration, making it challenging to define the levels of integration that impact the process. The review also did not identify factors beyond clinical boundaries that could hinder integration, such as stock-outs of contraception commodities. Lastly, social determinants were not within the scope of this review, so they were not explored.
8. **Involvement of individual with lived experience and practitioners:** This review did not directly involve individuals with lived experience of accessing integrated FP services, nor were frontline practitioners engaged in the design or interpretation of the findings. While the included studies reflect provider and client perspectives as reported in the primary data and qualitative data, we acknowledge that the absence of direct involvement of service users and implementers in this review process is a limitation. The inclusion of community representative, users and practitioners could have enriched the interpretation of contextual and experiential aspects of integration. Future work will benefit from co-design methodologies and closer engagement with those delivering and receiving care.

## Conclusion and the way forward

Integrating FP with reproductive health services in low-resource settings requires clear, context-sensitive frameworks. Advancing this agenda demands flexible integration models, investments in workforce training, co-located infrastructure, and follow-up systems. Future research should incorporate system-level and community perspectives, particularly those of women, youth, and frontline workers, to promote equitable and sustainable integration. These steps are essential to realise the full potential of integrated FP services in improving reproductive health outcomes in LMICs.

To optimise the integration of FP services with other reproductive health services in LMICs, a clearer articulation of integration processes is essential, grounded in both operational and contextual realities. Health systems should adopt standardised frameworks that allow flexible implementation across service platforms, supported by robust provider training, private counselling infrastructure, and tracking mechanisms for follow-up care. Beyond service delivery, future research must explore community engagement strategies and address systemic barriers such as supply chain gaps and gender-based stigma. Engaging both providers and clients in co-designing integration approaches will help ensure services are responsive, equitable, and sustainable.

## Additional File

The additional file for this article can be found as follows:

10.5334/ijic.8912.s1 Supplementary File. Supplementary Tables 1 to 6.
