# Supplementary material for: The Process of Integrating Family Planning Services with Other Reproductive Health Services in Low and Middle-Income Countries: A Scoping Review
Source: Int J Integr Care. 2025 Jul 1;25(3):2. doi: 10.5334/ijic.8912 (PMC12227082; doi:10.5334/ijic.8912)
Supplement: Supplementary File. — Supplementary Tables 1 to 6. [file ijic-25-3-8912-s1.pdf]

Supplementary Table 1. Inclusion Criteria Using PICCOS Framework

Component	Criteria	Examples from Included Studies
**P – Population**	Women of reproductive age in **low- and middle-income countries (LMICs)** accessing health services.	• Postpartum women in **Kenya** discharged without FP counselling [5] • HIV-positive women accessing ART clinics in **Ethiopia and Zambia** [37, 50]
**I – Intervention**	Integration of **family planning (FP)** services with **other reproductive health services**, such as: • Antenatal care (ANC) • Postnatal care (PNC) • Post-abortion care (PAC) • HIV/AIDS services • Immunisation services	• FP integrated with HIV services in **Tanzania and Kenya**, promoting dual method use [29, 34] • FP with immunisation in **Liberia and Nigeria**, using vaccinators to counsel and refer [39, 66] • FP offered alongside PAC in **Nepal and Bangladesh** using LARC counselling [32, 58]
**C – Comparison**	Studies with or without a comparator group; inclusion was not limited by presence of a control or comparison group.	• Non-randomised intervention in **Kenya** without a comparator [38] • Cluster RCT in **Rwanda** comparing integrated vs non-integrated models [39]
**C – Context**	Facility-based integration models where FP services are delivered: • In the same unit (same-room) • In different units but co-located within the same facility • Through internal referral mechanisms Excluded: community-only models, integration defined as referral alone	• Co-located FP and HIV services in **Uganda and Botswana** [41, 42] • ANC and FP provided in different rooms in **India** [47]
**O – Outcomes**	Focus on **integration processes**, such as: • Provider training • Referral arrangements • Client contact strategies • Link to FP outcomes (e.g., uptake, dual method use, unmet need)	• Use of peer educators to promote dual method in HIV clinics in **Zambia** [50] • Job aids and group counselling in immunisation settings in **Ghana and Pakistan** [39, 52] • Increased PPIUD uptake after ANC/PNC integration in **Nepal** [58]
**S – Study Design**	Empirical studies using: • Quantitative methods (e.g., trials, surveys) • Qualitative methods (e.g., interviews, FGDs) • Mixed-methods Excluded: reviews, protocols, opinion pieces, and training-only papers without integration focus	• Qualitative study exploring provider barriers in **Burkina Faso** [61] • Cluster RCTs measuring contraceptive outcomes in **Kenya and Rwanda** [34, 39] • Mixed-methods implementation evaluation in **Malawi** [35]

Supplementary Table 2. Overview of Study Characteristics

Author (Year)	Country	Service Integrated With FP	Study Design
Baumgartner et al. (2014)	Tanzania	HIV	Quantitative
Baumgartner et al. (2012)	Kenya	HIV	Quantitative
Baynes et al. (2022)	Tanzania, Zanzibar	Post-abortion	Quantitative
Biswas et al. (2017)	Bangladesh	PAC/MR	Quantitative
Chen et al. (2020)	Kenya	HIV	Quantitative
Cohen et al. (2017)	Kenya	HIV	Quantitative
Cooper et al. (2020)	Malawi	Immunisation	Mixed methods
Cooper et al. (2017)	Kenya	Nutrition	Mixed methods
Demissie & Mmusi-Phetoe (2021)	Ethiopia	HIV	Quantitative
Dulli et al. (2019)	Kenya	HIV	Quantitative
Dulli et al. (2016)	Rwanda	Immunisation	Quantitative
Erhardt-Ohren et al. (2020)	Benin	Immunisation	Mixed methods
Grossman et al. (2013)	Kenya	HIV	Quantitative
Hawkins et al. (2021)	Botswana	HIV	Quantitative
Ijarotimi et al. (2023)	Nigeria	Immunisation	Quantitative
Joshi et al. (2016)	India	HIV	Quantitative
Kirunda et al. (2017)	Uganda	HIV	Mixed methods
Kosgei et al. (2011)	Kenya	HIV	Quantitative
Mackenzie et al. (2018)	India, Kenya	ANC/PNC	Mixed methods
Malama et al. (2020)	Zambia	HIV	Quantitative
McGinn & Irani (2019)	Malawi	HIV	Quantitative
Medley et al. (2023)	Zambia	HIV	Quantitative
Mekonnen & Roets (2020)	Malawi	HIV	Qualitative
Memon et al. (2023)	Pakistan	MCH	Quantitative
Mudiope et al. (2017)	Uganda	HIV	Quantitative
Mutisya et al. (2019)	Kenya	MCH	Mixed methods
Nelson et al. (2019)	Liberia	Immunisation	Quantitative
Newmann et al. (2016)	Kenya	HIV	Quantitative
Olakunde et al. (2021)	Nigeria	HIV	Quantitative
Pradhan et al. (2019)	Nepal	Postpartum	Quantitative
Sheahan et al. (2021)	Nigeria	Immunisation	Quantitative
Tawfik et al. (2014)	Afghanistan	Postpartum	Mixed methods
Tougri et al. (2022)	Burkina Faso, Côte d'Ivoire, Niger	Postpartum	Qualitative
Thyda et al. (2015)	Cambodia	HIV	Quantitative
Vance et al. (2014)	Ghana, Zambia	Immunisation	Quantitative
Yugbaré Belemsaga et al. (2018)	Burkina Faso	Postpartum	Quantitative
Zewdie et al. (2020)	Ethiopia	HIV	Quantitative

Supplementary Table 3. Definitions of Integration in Selected Studies

Study	Definition of Integration	Service Context
Baumgartner et al., 2014	Provision of FP and HIV services on the same day, at the same facility and during the same operating hours—though possibly at different locations within the facility and by different providers.	FP + HIV services
Belemsaga et al., 2018	Services offered either at the same location or scheduled for the same day, a later day, or provided via referral mechanisms.	FP + maternal/postpartum services
Memon Z. et al., 2023	Integration defined across three dimensions: (1) Physical—services available in the same room/facility; (2) Temporal—services offered more than once a week; and (3) Functional—services aligned to the primary reason for the client's visit at each contact point.	FP + maternal/child health services
Zewdie et al., 2020	Services integrated within the same facility and delivered by different cadres of staff as part of routine care.	FP + HIV services
Thyda et al., 2015	Integration model with services available on the same day and at the same facility, though not necessarily at the same time.	FP + HIV services

Supplementary Table 4. Human Resource Capacity Building for Family Planning Integration

Author	Type of Provider	Training Provided On	Duration of Training	Training Material Used
Thyda et al., 2015	Doctor, midwife	FP services	Not mentioned	Not discussed
Malama et al., 2020	Counsellors, nurses	Counselling, dual method use, LARC	2–3 days	Zambian Ministry of Health model
Medley et al., 2022	Nurses, counsellors	Contraception, counselling	Not mentioned	Not discussed
Baumgartner et al., 2012	VCT providers	Screening, counselling, referral	Not mentioned	Not discussed
Demissie & Mmusi-Phetoe, 2021	Not mentioned	Not discussed	Not discussed	Not discussed
Grossman et al., 2013	Peer educators, HIV staff	Group education, FP	Not discussed	Not discussed

		interest		
Cohen et al., 2017	Peer educators, HIV staff	Group education, counselling, method insertion	3 days + 2-day practicum	Not discussed
Hawkins et al., 2021	Peer educators	Dual protection methods	Not mentioned	Not discussed
Baumgartner et al., 2014	HIV & FP providers	Referral, FP screening, counselling	Not mentioned	WHO MEC guideline
Joshi et al., 2016	Service provider	Dual protection methods	1 day + follow-up	WHO guideline
Kirunda et al., 2010	HIV care provider	Group counselling	Not mentioned	WHO counselling tool
Mutisya et al., 2019	Service provider	Training evaluated	Not specified	Not specified
Cooper et al., 2015	Vaccinators, FP provider	Messages and referral (vaccinator); postpartum FP (provider)	3 days	Not mentioned
Baynes et al., 2022	PAC provider	LARC	Not mentioned	Centralised PAC/LARC curriculum
Biswas et al., 2017	Doctors, midwives, nurses	PAC services	Not mentioned	WHO MVA/PAC manual
Belemsaga et al., 2018	Facility health workers	Postpartum care	Not mentioned	Not mentioned
Pradhan et al., 2019	Maternity care providers	PPIUD counselling and insertion	3-day workshop	Designed training guideline
Tawfik et al., 2014	FP counsellors	PPFP counselling	Not mentioned	Not mentioned
Mackenzie et al., 2018	Not mentioned	Not discussed	Not discussed	Not discussed
Sheahan et al., 2021	Health facility provider	FP counselling and provision	Not mentioned	Not mentioned
Cooper et al., 2020	Health surveillance assistant	LAM, pills, condoms, injectables	Not mentioned	Not mentioned
Dulli et al., 2016	Immunisation and FP providers	PPFP, screening	3 days + 1-day refresher	ESD & ACCESS-FP
Vance et al., 2014	Vaccinators and FP providers	Risk screening, referral	Not mentioned	Not mentioned
Nelson et al., 2019	Vaccinators	Message, referral to co-located services	3 days	Not mentioned
Ijarotimi et al., 2023	Vaccinators	FP education	Not mentioned	Not mentioned

Supplementary Table 5. Facilitating and Hindering Factors for Family Planning Integration

Author	Facilitating Factors	Hindering Factors
Ijarotimi et al., 2023	Training providers increased confidence to integrate FP into routine services.	Lack of refresher training; last FP training dated back to 1995.
Tougri et al., 2022	Providing services at the same location and on the same day helped reach postpartum women who might not return for FP.	Overburdened staff managing multiple services hindered integration effectiveness.
Nelson et al., 2019	Offering same-day services and tracking referrals helped increase FP uptake.	Lack of privacy during FP counselling discouraged some clients from participation.
Newmann et al., 2016	Integration created opportunities for group counselling and more equitable attitudes toward FP.	Gender dynamics and lack of male engagement limited the effectiveness of integrated services.
Sheahan et al., 2021	Strong provider-client interaction and staff willingness to integrate services enabled service continuity.	Inconsistent training coverage and staff turnover disrupted service continuity.
Mutisya et al., 2019	Provider-initiated FP discussions during ANC and PNC improved access and uptake of contraceptive services.	Logistical constraints in coordination between services delayed service delivery.

Supplementary Table 6. Linking Integration Strategies to Family Planning Outcomes

Author	Integration Strategy	Reported FP Outcome
Chen et al., 2020	FP integration into HIV treatment centres with trained staff and service alignment.	Higher contraceptive uptake among HIV clients.
Demissie & Mmusi-Phetoe, 2021	Counselling and referral for dual method use during ART visits.	Increased dual method use among ART clients.
Dulli et al., 2019	Peer educator-led FP education for female sex workers during HIV prevention services.	Improved FP uptake among female sex workers.
Joshi et al., 2016	FP and HIV service integration using trained staff for dual protection promotion.	Improved use of dual protection methods.
Medley et al., 2022	Health talks, fertility intention screening, and private service provision in HIV clinics.	Enhanced contraceptive access and uptake.
Pradhan et al., 2019	Integrated counselling and immediate PPIUD insertion during ANC/PNC and delivery.	High acceptance of PPIUD immediately after delivery.

Mudiope et al., 2017	FP champion-led counselling and escorted referrals in HIV clinics.	Increased FP uptake among HIV-positive mothers.
Baynes et al., 2022	Postabortion integration using facility-level training and centralised service provision.	Improved LARC uptake post-abortion.
Biswas et al., 2017	PAC services integrated with LARC counselling and referrals.	Greater PAC method acceptance.
Cohen et al., 2017	Group FP counselling and dual method education in HIV clinics.	Reduction in unintended pregnancies.
Grossman et al., 2013	HIV clinical staff trained to deliver group FP counselling and services.	Significant increase in FP use post-counselling.
Kosgei et al., 2011	HIV-FP service integration assessed for pregnancy outcome impact.	No significant impact on unintended pregnancy.
Mutisya et al., 2019	Provider-initiated FP discussions during ANC/PNC with service flow redesign.	Improved access to FP during MCH service flow.
Baumgartner et al., 2014	Referral-based FP integration in HIV care facilities.	Improved referral and counselling coverage.
Vance et al., 2014	FP messaging integrated with immunisation visits; referrals tracked.	Increase in FP referrals and uptake.
Ijarotimi et al., 2023	FP education at infant vaccination visits with group and individual counselling.	Increased postpartum contraceptive use.
